# The Warburg Effect Is Genetically Determined in Inherited Pheochromocytomas

**DOI:** 10.1371/journal.pone.0007094

**Published:** 2009-09-18

**Authors:** Judith Favier, Jean-Jacques Brière, Nelly Burnichon, Julie Rivière, Laure Vescovo, Paule Benit, Isabelle Giscos-Douriez, Aurélien De Reyniès, Jérôme Bertherat, Cécile Badoual, Frédérique Tissier, Laurence Amar, Rosella Libé, Pierre-François Plouin, Xavier Jeunemaitre, Pierre Rustin, Anne-Paule Gimenez-Roqueplo

**Affiliations:** 1 INSERM, unit 970, Paris, France; 2 Collège de France, Paris, France; 3 Université Paris Descartes, Faculté de Médecine, Paris, France; 4 Paris-Cardiovascular research Center at HEGP, Paris, France; 5 INSERM, unit 676, Hôpital Robert Debré, Paris, France; 6 Université René Diderot, Faculté de Médecine, Paris, France; 7 Programme Cartes d'Identité des Tumeurs, Ligue Nationale Contre Le Cancer, Paris, France; 8 AP-HP, Hôpital Cochin, Département d'Endocrinologie, Paris, France; 9 AP-HP, Hôpital Européen Georges Pompidou, Service d'Anatomo-pathologie, Paris, France; 10 AP-HP, Hôpital Cochin, Service d'Anatomie pathologique, Paris, France; 11 AP-HP, Hôpital Européen Georges Pompidou, Service d'Hypertension artérielle, Paris, France; 12 AP-HP, Hôpital Européen Georges Pompidou, Département de Génétique, Paris, France; 13 Rare Adrenal Cancer Network-Corticomédullosurrénale Tumeur Endocrine, Institut National du Cancer, Paris, France; City of Hope Medical Center, United States of America

## Abstract

The Warburg effect describes how cancer cells down-regulate their aerobic respiration and preferentially use glycolysis to generate energy. To evaluate the link between hypoxia and Warburg effect, we studied mitochondrial electron transport, angiogenesis and glycolysis in pheochromocytomas induced by germ-line mutations in *VHL*, *RET, NF1* and *SDH* genes. SDH and VHL gene mutations have been shown to lead to the activation of hypoxic response, even in normoxic conditions, a process now referred to as pseudohypoxia. We observed a decrease in electron transport protein expression and activity, associated with increased angiogenesis in *SDH-* and *VHL-*related, pseudohypoxic tumors, while stimulation of glycolysis was solely observed in VHL tumors. Moreover, microarray analyses revealed that expression of genes involved in these metabolic pathways is an efficient tool for classification of pheochromocytomas in accordance with the predisposition gene mutated. Our data suggest an unexpected association between pseudohypoxia and loss of p53, which leads to a distinct Warburg effect in *VHL*-related pheochromocytomas.

## Introduction

In 1930, the biochemist Otto Warburg proposed that cancer was caused by defects in oxidative phosphorylation (OXPHOS) or aerobic respiration in the mitochondria; this would force the cell to shift to an anaerobic energy generation process, glycolysis, despite aerobic conditions [1], [2]. After being forgotten for decades, the Warburg effect is being reconsidered and is now the subject of increasing interest and analysis [3], [4], [5]. OXPHOS and glycolysis have been evaluated in numerous tumor types, including renal clear cell carcinoma (RCC) in which the majority of glycolytic enzymes are overexpressed and mitochondrial enzymes underexpressed relative to patient-matched normal kidney cortex [6]. The Von Hippel-Lindau (*VHL*) gene is inactivated in 80% of sporadic RCC, and was thus suspected to mediate this phenomenon. *VHL* is a tumor suppressor gene responsible for the VHL disease, a hereditary neoplastic syndrome characterized by a predisposition to RCC, retinal and central nervous system hemangioblastomas, pancreatic cysts and pheochromocytomas [7]. *In vitro* studies confirmed that the presence of the VHL protein was required for intact respiratory chain protein content and activities in RCC [8], [9].

Pheochromocytomas (PH) or functional paragangliomas (fPGL) are rare catecholamine-secreting tumors arising from the adrenal medulla or sympathetic nervous ganglia. Approximately 25–30% of these tumors occur in the context of a hereditary cancer syndrome [10], one third of which are caused by mutations in the *VHL* gene. The other forms are mediated by mutations in the *RET* proto-oncogene and the *NF1*, *SDHB*, *SDHC*, or *SDHD* tumor suppressor genes. *SDHB, C* and *D* genes encode three of the four subunits of succinate dehydrogenase (SDH), a mitochondrial enzyme, which catalyzes the oxidation of succinate into fumarate in the tricarboxylic acid (TCA) cycle, and feeds electrons to the ubiquinone pool in the respiratory chain. Identification of mutations in *SDH* genes led to the first and unexpected demonstration of a tumor suppressor role for a metabolic enzyme (for review see [11], [12]), implicating mitochondrial deficiencies in tumorigenesis, as first suggested by Otto Warburg 80 years earlier.

Transcription profiling of hereditary and sporadic primary PH/PGL revealed that tumors associated with *VHL* [13], and later *SDH* [14] mutations display diverse angiogenesis and hypoxia markers and reduced expression of components of the oxidative response and TCA cycle. A hypoxia-inducible transcription factor (HIF)-dependent decrease in SDHB protein expression has also been described, specifically in *SDH-* and *VHL*-related PH/PGL.

A common feature of *SDH* and *VHL* mutations is their capacity to mediate a pseudo-hypoxic response, *i.e.* the abnormal stabilization of HIFs under normoxic conditions. The pVHL protein is an E3 ligase recognition factor, and its key function is the ubiquitination and subsequent degradation of the α subunit of HIF-1 and -2 in normoxia [7]. Inactivation of SDH also leads to HIF stabilization, through the inhibition of their hydroxylation by prolyl-4-hydroxylases, necessary for their recognition by pVHL [15], [16]. HIFs may be important for the molecular modulation of the Warburg effect: HIF-1α is a key regulator of glycolysis and induces the expression of glucose transporters, glycolytic enzymes and lactate dehydrogenase; it also mediates the expression of pyruvate dehydrogenase kinase 1, which inhibits the conversion of pyruvate to acetyl-CoA, thereby attenuating mitochondrial function and respiration (for review, see [17]).

In this study, we investigated whether there is an increased Warburg effect in *VHL*-related PH/PGL, mediated by the pseudo-hypoxic drive. We compared angiogenesis, mitochondrial metabolism and glycolysis in PH/PGL tissues from patients suffering from VHL disease and patients presenting mutations affecting the *SDH, RET* or *NF1* genes.

## Results

### Evaluation of HIFs Expression and Angiogenesis in PH/PGL

**Figure 1:**
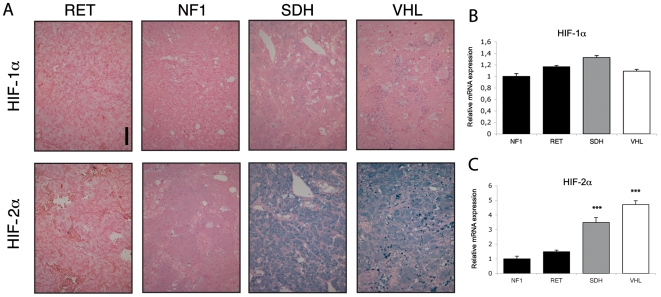
Pseudohypoxia in SDH and VHL-related PH/PGL. (A) HIF-1α and HIF-2α immunohistochemistry were performed to evaluate activation of the hypoxic pathway in all samples. Histogreen was used as a chromogen for detection (blue labeling). Calibration bar: 100 µm. Microarray evaluation of HIF-1α (B) and HIF-2α (C) expression between SDH, VHL, RET and NF1 tumors. Data are means±SEM. ***p<0.001.

First, we performed HIF-1α and HIF-2α immunohistochemistry to evaluate pseudohypoxia in inherited PH/PGL tissues (Fig. 1A). We did not detect the expression of either HIF-1α or HIF-2α in RET and NF1 tumors, although both these proteins were present in adjacent adrenal tissues (data not shown). A very weak HIF-1α nuclear labeling was observed in 5 out of 8 SDH-related and in only 1 out of 10 VHL-related tumors studied. In contrast, HIF-2α was expressed at a much higher level both in the nucleus and cytoplasm of tumor cells from all 8 SDH samples and 7 out of 10 VHL PH/PGL. An increase in HIF-2α mRNA expression has previously been reported in VHL PH/PGL [13]. We thus compared the expression of both HIFs using genome-wide expression micro-array in a population of 68 inherited PH/PGL (28 VHL, 9 NF1, 9 RET, 17 SDHB, 3 SDHD and 2 SDHC) (Fig. 1B and C). We observed that HIF-2α was indeed overexpressed in VHL and in SDH-related tumors, as compared to RET and NF1 ones (4.7 (p = 10^−6^) and 3.5-fold (p = 10^−5^) increase vs NF1, respectively). There was no difference in HIF-1α mRNA levels between the different types of tumors studied.

**Figure 2:**
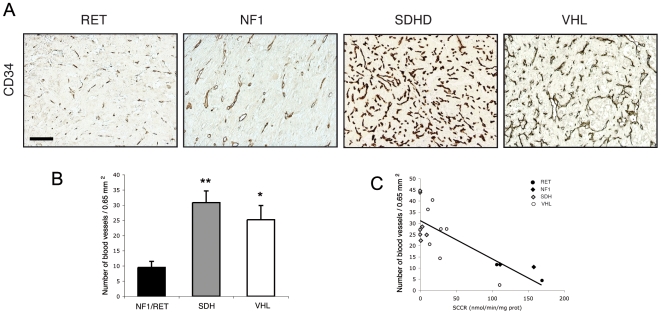
Angiogenesis in SDH and VHL-related PH/PGL. (A) CD34 immunohistochemistry was performed to evaluate angiogenesis in all samples. Diaminobenzidin was used as a chromogen for detection (brown labeling). Calibration bar: 200 µm. (B) Quantification of vascular density showing an increased number of blood vessels in SDH, and VHL tissues. Data are means±SEM. *p<0.05, **p<0.01, ***p<0.001. (C) Correlation between vascular density and SCCR enzymatic values for individual patients.

We then used CD34 immunohistochemistry to evaluate the vascular density in PH/PGL samples (Fig. 2A). Blood vessel density was 2.5 to 3.5-fold higher in VHL (p<0.05) and in SDH (p<0.01) related tumors than in RET and NF1-related tumors (Fig. 2B).

### Expression and Activity of the OXPHOS Complexes

**Figure 3:**
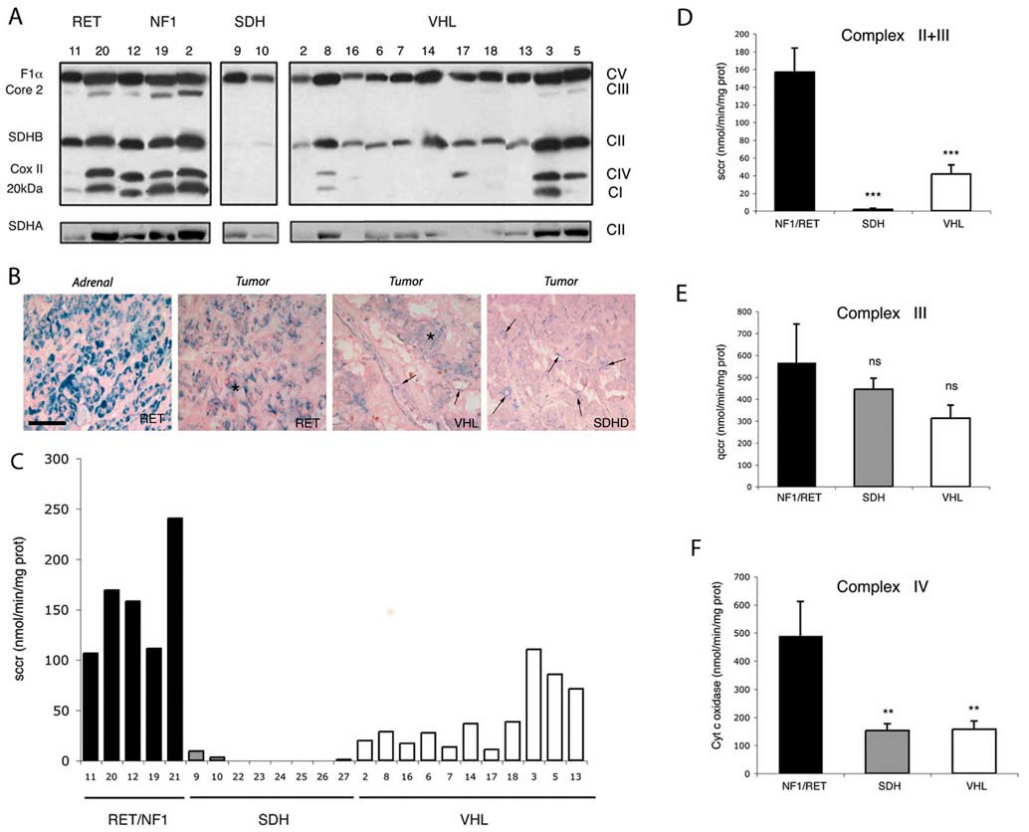
Decreased oxidative phosphorylation in SDH and VHL-related PH/PGL. (A) The abundance of proteins of mitochondrial complexes I (20 kDa subunit), II (SDHB and SDHA), III (Core 2) and IV (Cox II) is lower in PH/PGL from SDH and most VHL than from RET and tumor tissues. (B) SDHB immunohistochemistry performed on the adrenal adjacent to a RET-related PH and in RET, VHL and SDHD-mutated PH reveals a strong labeling in the adrenal compared to tumor cells (asterisks). In VHL PH/PGL tumor, expression of SDHB was reduced when compared to RET-related PH while it is absent in SDHD-related tumor. Note that vascular immunostaining was present in all samples (arrows). Calibration bar: 50 µm. (C) Individual values of SCCR activity reveal that low complex II+III enzymatic activity is associated with low protein abundance. (D–F) Mean values for mitochondrial complexes II+III, III and IV reveal a generalized decrease in respiration in SDH and VHL PH/PGL. Data are means±SEM. **p<0.01, ***p<0.001.

We then tested for the Warburg effect in inherited PH/PGL. We first analyzed the expression and activity of various complexes of the mitochondrial electron transport system by Western blotting using a cocktail of monoclonal antibodies optimized for the detection of one subunit of each of the five OXPHOS complexes (20 kDa subunit in Complex I, SDHB in Complex II, core 2 in Complex III, COX II in Complex IV and F1α in ATP synthase). Each subunit identified was found to be less abundant when the corresponding complex failed to assemble properly. SDHA protein was assayed independently in all PH/PGL. Proteins of complexes I to IV were less abundant in *SDH-* and *VHL*-mutated tumors than in PH/PGL harboring *NF1* and *RET* mutations, but complex V was relatively similar in all patients (Fig. 3A). Using immunohistochemistry, we confirmed our recently described observation that SDHB protein expression was completely lost in tumor cells of SDH-related patients and noticeably reduced in VHL-tumors, while it was still present in vascular endothelial and smooth muscle cells (Fig. 3B) [18].

Succinate cytochrome *c* reductase activity (SCCR, complex II + III) was measured in tumor homogenates: individual SDHB and SDHA protein expression followed SDH enzymatic activity. Complex II+III electron transfer activity was abolished in SDH and substantially lower in VHL samples than in NF1 and RET tumors (Fig. 3C). The mean values of enzymatic activities revealed that SCCR activity was 80-fold lower in SDH tumors (p<0.001) and 4-fold lower in VHL (p<0.01) tumors than in RET and NF1 samples (Fig. 3D). Interestingly, SCCR enzymatic activity as measured on these frozen tumor tissues was inversely correlated to the vascularization quantified in the corresponding paraffin-embedded samples (Fig. 2C). A low complex II activity correlated with a high vascular density (n = 18; r = −0.71; p<0.001).

Biochemical studies also revealed that cytochrome c oxidase (COX, complex IV) activity was approximately 3-fold lower in SDH (p<0.01) and in VHL (p<0.01) samples than in NF1 and RET tumors (Fig. 3E). Finally, we assayed quinol cytochrome C reductase (QCCR, complex III) activity in tumor homogenates. QCCR activity was also slightly lower in SDH and VHL tumors, but the difference was much smaller (1.3 and 1.8-fold lower, respectively, than in NF1 and RET tumors) and was not statistically significant (Fig. 3F).

### Induction of Anaerobic Glycolysis in VHL-related PH/PGL

**Figure 4:**
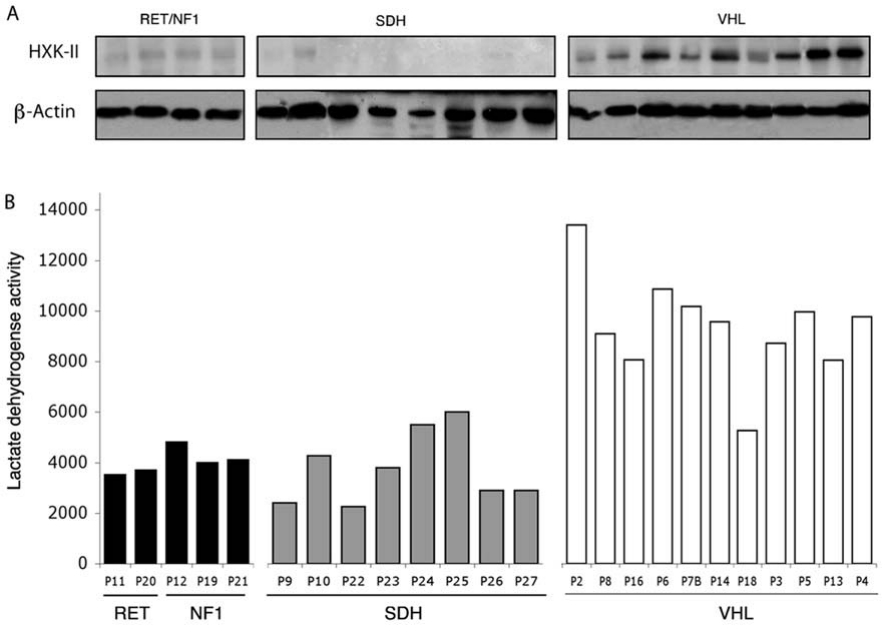
Increased glycolysis in VHL-related PH/PGL. (A) Hexokinase-II protein is detected in all VHL PH/PGL but is hardly detectable in RET, NF1 and SDH samples. (B) Lactate dehydrogenase is increased in tumors harboring a VHL mutation as compared to SDH, RET and NF1 tumors.

We then evaluated several aspects of anaerobic glycolysis in tumor tissues. Activation of the HIF pathway in SDH and VHL PH/PGL may be responsible for the stimulation of glycolysis and anaerobic fermentation. Using immunohistochemistry, we studied the glucose transporter Glut1, a well-described HIF-1α target, but found that it was restricted to erythrocytes, and was not expressed in chromaffin cells within PH/PGL tissues (data not shown). We then assessed the expression of hexokinase-II (HXK-II), another HIF-1 target, responsible for the first step of glycolysis, *i.e*. glucose phosphorylation into glucose-6P. HXK-II is the predominant hexokinase isoform overexpressed in cancers that exhibit a Warburg effect [19]. Surprisingly, we observed that HXK-II was expressed in all VHL samples but was at the threshold of detection in all RET, NF1 and SDH tumors (Fig. 4A). Likewise, we found that lactate dehydrogenase (LDH), which catalyses the last step of anaerobic glycolysis and can be used as an indicator of glycolytic activity [4], was twice as active in all VHL tumors (p<0.01) than in NF1 and RET PH/PGL. LDH activity in SDH tissues was similar to that in RET and NF1 samples (Fig. 4B) and clearly distinguished VHL from non-VHL PH/PGL (p = 0.001).

### Microarray Evaluation of Electron Transport and Glycolysis-Related Genes in Inherited PH/PGL

**Figure 5:**
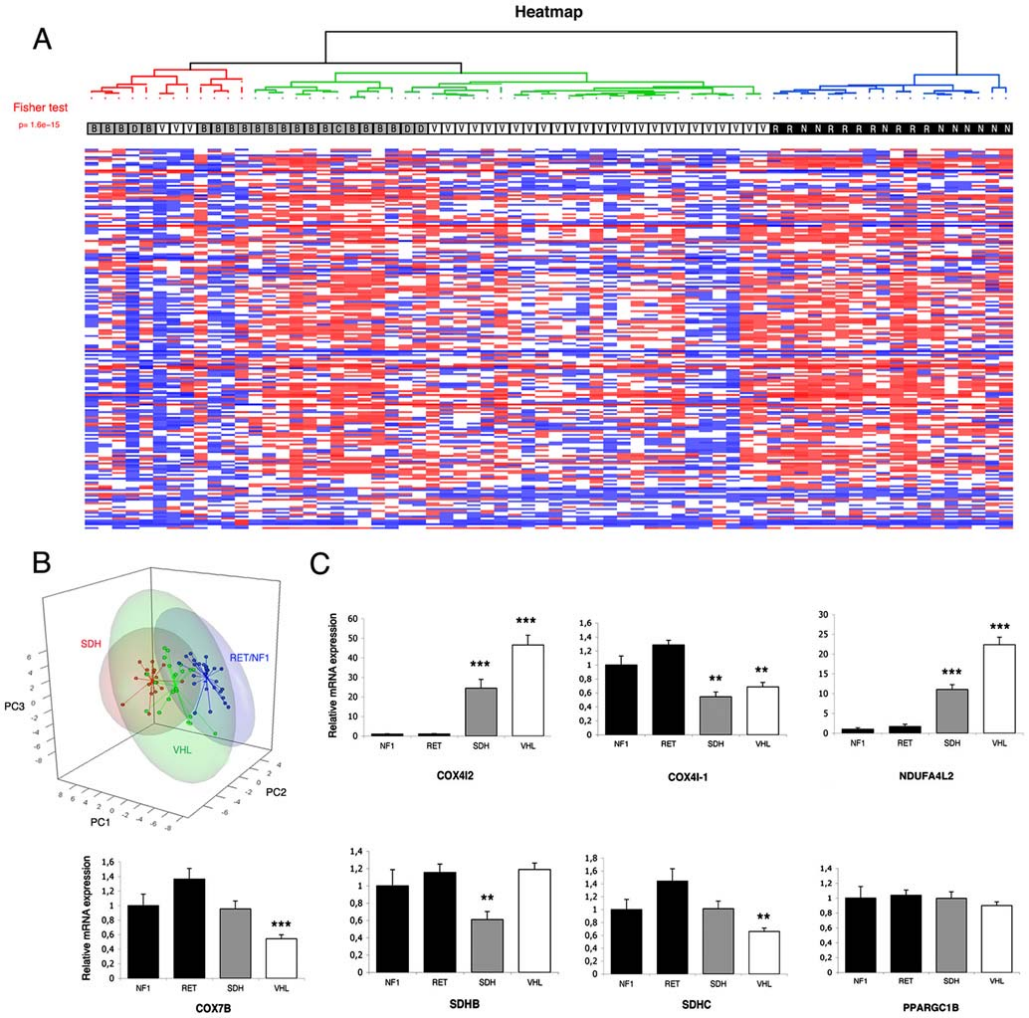
Microarray analysis of oxidative phosphorylation in hereditary PH/PGL. (A) Unsupervised hierarchical clustering analysis of the 68 samples according to the expression of 200 genes. Expression profiles are shown as a heat map indicating high (red) and low (blue) expression according to a log2-transformed scale. The higher bipartition allows to distinguish VHL (white) and SDH (grey) patients from RET and NF1 (black) patients. (B) Principal component analysis of the 68 samples according to the expression of 200 genes. Three groups are focused on, corresponding to the SDH (red), VHL (green) and RET/NF1 (blue) patients. PC1: principal component; PC2: principal component 2; PC3: principal component 3. (C) Mean values for genes expression between SDH, VHL, RET and NF1 tumors. Data are means±SEM, represented as relative to NF1 expression values. **p<0.01, ***p<0.001.

In order to validate these observations in a larger cohort, we performed genome-wide expression microarray analysis in a population of 68 inherited PH/PGL. We compared the expression profile of genes involved in OXPHOS in 28 VHL tumors with that of 40 non-VHL PH/PGL (9 NF1, 9 RET, 17 SDHB, 3 SDHD and 2 SDHC). Among the 127 genes studied (200 probe sets), 47 genes (57 probe sets) were differentially expressed in VHL samples (Welch's t-test p<0.01). With the exception of two genes (COX4I2 and NDUFA4L2), all genes were down-regulated in the VHL group. The expression profile of these 200 probe sets was then used to perform a clustering of the 68 tumors (Fig. 5A). Such an analysis allowed classifying patients into two groups; the first one included all VHL and SDH patients and the second one included all RET and NF1 patients Interestingly, the first cluster could be divided into three subclusters, which separated SDH tumors in one hand, and VHL PH/PGL in the other hand. The expression profile of these 200 probe sets was also used to perform a principal component analysis on the 68 tumors and led to classification of patients into three groups: SDH, VHL and RET/NF1 tumors (Fig. 5B).

Reductions in gene expression detected by microarray analysis were subtle, and differences never reached a two-fold decrease. In contrast, the two increased genes were strongly upregulated. COX4I2 was overexpressed 25- and 45-fold in SDH and VHL tumors vs NF1, respectively. NDUF4L2 was increased by 11- and 21-fold in SDH and VHL tumors vs NF1, respectively (Fig. 5C).

**Figure 6:**
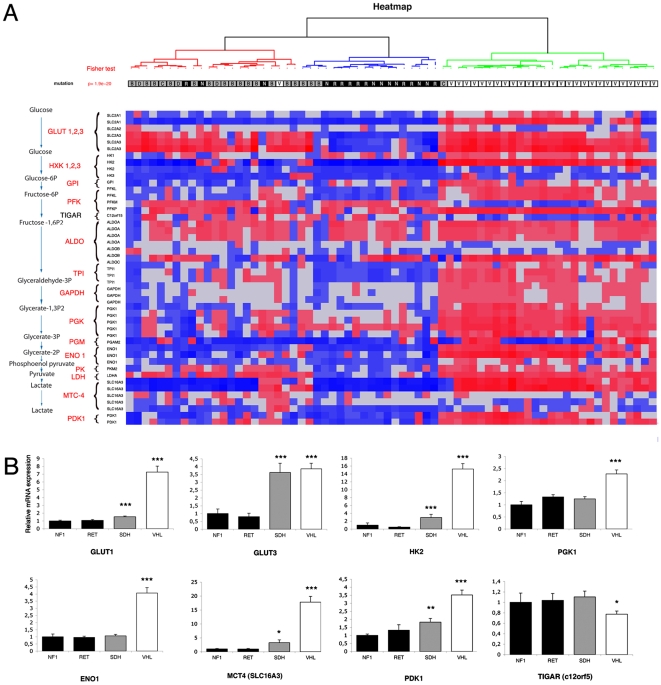
Microarray analysis of glycolysis in hereditary PH/PGL. (A) Unsupervised hierarchical clustering analysis of the 68 samples according to the expression of 38 genes. Expression profiles are shown as a heat map indicating high (red) and low (blue) expression according to a log2-transformed scale. The different mutations are localized in three distinct clusters: SDH (grey), VHL (white) and RET/NF1 (black). (B) Mean values for genes expression between SDH, VHL, RET and NF1 tumors. Data are means±SEM, represented as relative to NF1 expression values. **p<0.01, ***p<0.001.

We performed the same analysis with genes involved in the glycolytic pathway. Among the 20 genes studied (38 probe sets), 19 genes (37 probe sets) were differentially expressed in VHL samples (Welch's t-test p<0.01), including 16 upregulated genes (33 probe sets) in the VHL group. The expression profile of these 38 probe sets was used to perform a clustering of the 68 tumors and led to the division of patients into two clusters (Fig. 6A). The first one regrouped all non-VHL patients (SDH, NF1 and RET), while all VHL tumors were included in the second (except one VHL in the first cluster). Analysis of individual genes revealed a significant up-regulation of Glut1, Glut3, HXK-II, phosphofructokinase (PFK), enolase 1 (eno1), phosphoglycerate kinase 1 (PGK1), LDHA, pyruvate dehydrogenase kinase 1 (PDK1), monocarboxylate transporter 4 (MTC-4), phosphoglucomutase 1 (PGM1) in VHL versus non-VHL tissues. The average overexpression was between 2- to 4-fold, but reached 15- to 17-fold for HXK-II and the lactate transporter MTC-4, respectively. Interestingly, although SDH patients displayed levels of expression usually comparable to that observed in RET and NF1 tumors, Glut3, HXK-II and LDHA were also significantly overexpressed in this subset of patients (Fig. 6B).

### Loss of p53 and TIGAR Expression in VHL-related PH/PGL

Glycolysis was stimulated in all VHL samples but not in SDH tumors, and it appeared that stimulation of the HIF pathway could not account for this phenomenon. Recently, the tumor suppressor p53 was revealed to be a new and unexpected target, stabilized and activated by pVHL [20]. p53 is a transcription factor that regulates the expression of TIGAR (TP53-induced glycolysis and apoptosis regulator), a protein displaying fructose-2,6-bisphosphatase activity, thereby lowering fructose-2,6-bisphosphate concentrations in cells, resulting in inhibition of glycolysis [21]. These findings suggest that anaerobic glycolysis may be stimulated in cells that lack functional p53. Microarray analysis in the 68 inherited tumors revealed a modest but significant decrease of TIGAR mRNA expression (0.7 fold, Welch T-test p<0.05) in VHL vs non-VHL tumors (Fig. 6B).

**Figure 7:**
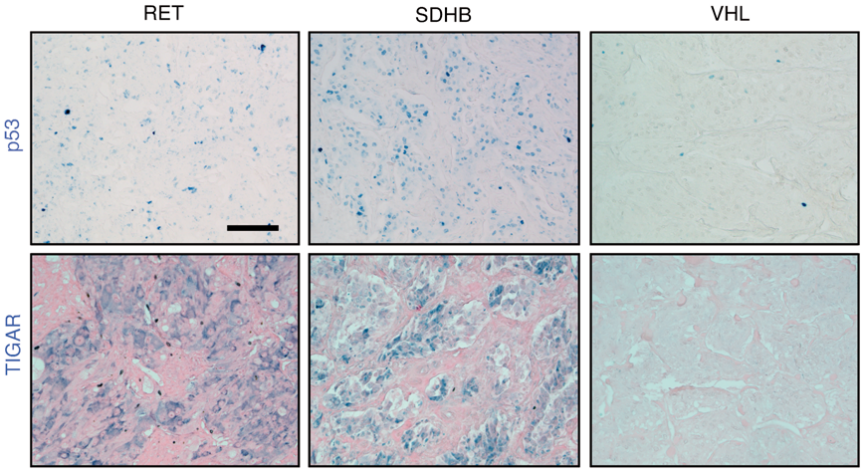
Representative expression of p53 and TIGAR in PH/PGL. Immunohistochemistry revealed a mild to strong nuclear p53 labeling, and the cystoplasmic expression of TIGAR in the majority of RET, NF1 and SDH tumors. Such labeling was only detected in 2 out of 9 VHL tumor tissues (data not shown). In most VHL PH/PGL p53 protein and its target TIGAR were hardly detectable. Calibration bar: 50 µm.

We thus evaluated the expression of p53 and TIGAR by immunohistochemistry in 20 available paraffin-embedded specimens (Fig. 7, Table 1). With the exception of 3 patients, cytoplasmic expression of TIGAR was systematically associated with the presence of p53 in the nucleus of tumor cells. Patients could be classified into two groups regarding TIGAR expression. A positive group displayed a widespread expression of TIGAR, either throughout the whole tumor sample or heterogeneously, with positive areas adjacent to TIGAR negative regions. A negative group showed no TIGAR labeling in tumor cells. The positive group comprised two NF1, one RET, six SDH and one VHL tumors. The negative group was composed of seven VHL, two SDH and one RET tumors. Loss of TIGAR expression thus appeared preferentially in *VHL*-related PH/PGL (p = 0.025).

**Table 1:** Expression of p53 and TIGAR in inherited PH/PGL evaluated by immunohistochemistry.

Patient n°	Gene mutated	TIGAR	p53
P11	RET	+	−
P20	RET	−	−
P12	NF1	+	+
P19	NF1	+	+
P9	SDHD	+	+
P10	SDHB	+	+
P22	SDHB	+	+
P23	SDHB	−	+
P24	SDHB	−	+
P25	SDHB	+	+
P26	SDHD	+	+
P27	SDHC	+	+
P6	VHL	−	−
P7	VHL	−	+
P14	VHL	−	−
P16	VHL	−	−
P18	VHL	−	−
P3	VHL	−	−
P4	VHL	−	−
P5	VHL	+	+

TIGAR is absent from 7 of 8 VHL tumor tissues, but was detected in 9 of 12 RET, NF1 and SDH tumors.

## Discussion

Here, we show that PH/PGL susceptibility genes influence the complex cellular balance between angiogenesis, electron transport and glycolysis, which are regulated by two master genes, HIF and p53. Our results suggest that activation of the HIF pathway associated with the loss of p53 activity might mediate the molecular and biochemical features of the Warburg effect; this effect was clearly observed in VHL PH/PGL, but not in RET and NF1 and only partially in SDH tumors.

In accordance with the observations reported by Dahia *et al* [14], we observed an overall down-regulation of SDHB protein level and activity in *SDH-* and *VHL*-mutated tissues, but this was not specific to the SDHB subunit as it was associated with comparable decreases in SDHA expression and in SDH activity. Activation of HIFs is a major aspect of cancer biology [22] and has been associated with the Warburg effect. In VHL-deficient RCC cell lines, it was recently shown that HIF-1αinhibits mitochondrial respiration by activating MXI-1 transcription, which encodes a c-Myc repressor [23]. C-Myc inhibition leads to down-regulation of PCG-1β, a PPARγ-coactivator that has been implicated in mitochondrial biogenesis [24]. We therefore studied the expression and activity of other complexes of the respiratory chain. We found an overall decrease in subunits of complexes I to IV in tumors from SDH and VHL patients. Biochemical analyses showed a reduction in enzyme activities affecting the cytochrome *c* oxidase (COX; complex IV) and, also, but to a much lesser extent, quinol cytochrome *c* reductase (complex III). Again, such defects were not observed in tumors that retain the ability to degrade HIFs in normoxia (*i.e*. RET and NF1 tumors). Using transcriptome analysis, we confirmed these findings and showed that unsupervised analysis of gene expression in the OXPHOS pathway is highly efficient to classify patients depending on cancer-predisposing gene. However, for genes that were significantly down-regulated in pseudo-hypoxic tumors, differences in mRNA expression were subtle, suggesting that regulation of mitochondrial respiration principally occurred at the post-traductional level. We performed MXI-1 and c-Myc immunohistochemistry (data not shown) and analyzed MXI-1 and PGC1-β mRNA expression by microarray, but such experiments led to negative results. There was no difference in their respective expression in the different types of hereditary PH/PGL, suggesting that the crosstalk between HIF and c-Myc pathways described in RCC or lymphoma cell lines [23], [25], was not implicated in the decreased mitochondrial biogenesis observed in human PH/PGL tissues.

Interestingly, a recent study showed that HIF-1 tightly regulates the expression of the COX4-1 and COX4-2 subunits and of the LON mitochondrial protease, in such a way that conditions stabilizing HIF-1 lead to a decreased COX activity, which was interpreted by the authors as an optimization of respiration efficiency in hypoxia [26]. The microarray analysis we performed on 68 inherited PH/PGL confirmed the observations made *in vitro* in human tumor tissues. COX4-2 was drastically overexpressed in pseudo-hypoxic PH/PGL as compared to RET and NF1 tumors, while COX4-1 was decreased two-fold. These results provide a possible molecular mechanism for the decrease in COX activity observed in SDH and VHL tumors. The NDUFA4L2 transcript was also strongly overexpressed in PH/PGL harboring SDH and VHL mutations. Interestingly, this gene was identified as the first gene up-regulated in a transcriptome analysis of hypoxic neuroblastoma cells, another neural-crest derived tumor that shares a number of features with PH/PGL [27]. NDUFA4L2 encodes a protein homologous to the NADH dehydrogenase (ubiquinone) 1 alpha subcomplex 4, a subunit of mitochondrial complex I. Although the function of this protein is unknown, one could postulate that it may act as a COX4-1/COX4-2 switch, limiting complex I activity in hypoxic tumors. Altogether, our observations demonstrate a reduced electron transport in some inherited PH/PGL and suggest that activation of the hypoxic response may be directly involved in the decreased mitochondrial respiration in these tumors.

One of the first described functions of the HIF pathway is activation of glycolysis; HIF-1α up-regulates the expression of most glycolytic enzymes and the glucose transporters GLUT1 and 3. As for vascularization and reduction in electron transport complexes, we thus expected to observe more active glycolysis in SDH and VHL PH/PGL than in RET and NF1 tumors. Most surprisingly, both expression of hexokinase II and the activity of lactate dehydrogenase were high in VHL patients, but not in SDH specimens. Transcriptome analysis confirmed the specific activation of glycolysis in VHL PH/PGL, indicating that such analysis was a powerful tool to discriminate VHL from non-VHL inherited PH/PGL. It is likely that in the case of an SDH defect, the cells might cope more easily with decreased ATP production by the mitochondria, as most of the respiratory chain will still be functional, and several metabolic shunts are potentially able to provide electrons and generate a membrane potential sufficient to generate normal levels of ATP. These observations were however unexpected, notably because positron emission tomography with 2-[(18)F]-fluoro-2-deoxy-D-glucose ([(18)F]-FDG PET) tracing glucose uptake was recently shown to be particularly sensitive for the evaluation of bone metastases in patients harboring *SDHB* mutations [28]. However, it is worth noting that, if whole glycolysis was not enhanced in SDH patients (as compared to RET or NF1), this subset of tumors did present a 4-fold increase in GLUT3 mRNA expression, as assessed by microarray. This augmentation would be sufficient to explain the high sensitivity of SDH tumors to [(18)F]-FDG PET and confirms that such approach is indeed relevant for the clinical evaluation of *SDHB*-related patients. The overexpression of mRNA encoding GLUT1 (7-fold), GLUT3 (4-fold) and HXK-II (15-fold) in VHL PH/PGL suggests that sensitivity for [(18)F]-FDG PET should be evaluated in these patients, where it is expected to be highly effective.

One possible explanation for the discrepancy observed between VHL and SDH tumors for the rest of the glycolytic pathway arises from the capacity of HIF-1α and HIF-2α to regulate the expression of different target genes: HIF-1α is the transcription factor involved in hypoxic induction of glycolysis but HIF-2α does not seem to participate in this process [29]. Pollard *et al.* recently reported the immunohistological analysis of HIFs factors in VHL and SDH-related PH/PGL [30]. They described a relatively higher expression of HIF-2α than HIF-1α in VHL tumors and a reverse pattern in SDH tumors. Surprisingly, in our subset of tumors, we observed high HIF-2α and low HIF-1α expression in both SDH and VHL PH/PGL. The reason for this discrepancy remains to be addressed and may be dependant on patients or, although unlikely, on experimental conditions. On the other hand, the predominance of HIF-2α is supported by previous observations performed in fetal paraganglia and neuroblastoma [31]–[33] and by its upregulation at mRNA level in both VHL and SDH PH/PGL. It could explain the absence of hypoxia-mediated activation of the glycolytic response in these tumors. It thus appears that the stimulation of glycolysis in VHL-PH/PGL is not fully mediated by activation of the HIF pathway, but also depends on an additional function of pVHL.

Recently, Roe *et al.* identified p53 as a new and unexpected target of pVHL [20]. Unlike its activity on HIF proteins, VHL binding to p53 suppresses Mdm2-mediated ubiquitination and nuclear export and thereby leads to p53 stabilization. pVHL also binds ATM and p300 to favor their stabilizing and activating properties on p53. Loss of p53 has recently been associated with mitochondrial and glycolytic metabolism. It can physically localize to mitochondria where it causes mitochondrial DNA depletion and altered reactive oxygen species homeostasis [34]. As a nuclear transcription factor, it activates the expression of SCO2 [35] and TIGAR [21], respectively implicated in the COX multimeric protein complex assembly required for OXPHOS and in inhibition of glycolysis. It therefore seemed plausible that p53-dependant loss of TIGAR in *VHL*-mutated samples may explain the activation of glycolysis specifically observed in these tissues. In normal cells, nuclear p53 expression is usually below the detection level of immunohistochemistry. Mutant p53, because of its longer half-life can result in positive nuclear immunostaining. In the present study, we were unable to detect p53 in PH/PGL samples using a standard immunohistochemistry protocol. This observation is in accordance with most reported studies that showed very little p53 mutations in benign PH/PGL [36]–[38]. However, using tyramide signal amplification, we were able to reveal p53 expression in tumor tissues. We confirmed that TIGAR protein was absent from 7/8 VHL-PH/PGL samples analyzed, but was present in 9/12 non-VHL samples. These observations are the first *in situ* demonstration of the link between VHL inactivation and loss of the p53-TIGAR pathway in a human tumor. They provide a pertinent explanation for the specific activation of glycolysis in VHL-related PH/PGL.

In conclusion, our data establish the occurrence of a Warburg effect specifically in PH/PGL harboring a *VHL* germ-line mutation. Analysis of the underlying mechanism suggests that this phenomenon depends on the activation of the pseudo-hypoxic pathway — most probably mediated by the abnormal stabilization of HIF-2α following VHL inactivation — and the loss of the p53/TIGAR pathway. Together, these phenomena concur to the observed decreased electron transport and increased glycolysis. In SDH tumors, where p53 loss is not expected nor detected, activation of whole glycolysis was not observed. Finally, in RET and NF1 tumors, which are neither related to an activation of the HIF pathway nor to a loss of p53, we did not observe any of the metabolic or angiogenic inductions discernible in VHL tumors. These results thus provide a direct insight into the mechanisms underlying tumorigenesis in VHL-related PH/PGL and their links with metabolic disorders.

## Materials and Methods

### Patients

We first analyzed 26 tumors collected by the COMETE network from 25 patients (11 men, 14 women) operated on between 1994 and 2005, in two tertiary referral centers in Paris: the Hypertension Unit in Hôpital Européen Georges Pompidou and the Department of Endocrinology in Hôpital Cochin. Twenty were pheochromocytomas and 6 were functional extra-adrenal paragangliomas. The procedures used for PH/PGL diagnosis and genetic testing were in accordance with institutional guidelines and have been described previously [10], [39]. The associated syndromic lesions and the family history of each case are described in Table 2. Two patients had a *NF1* phenotype. The genetic testing identified a germ-line *RET* mutation in three patients, an *SDHB* mutation in five patients, an *SDHD* mutation in two patients, an *SDHC* mutation in one patient and a *VHL* missense mutation in 12 patients. A large deletion in *VHL* from exon 1 to exon 3 was detected in one patient by the QMPSF method [39]. For each patient, we obtained two or three frozen samples and two paraffin-embedded specimens prepared for routine pathological analyses.

**Table 2:** Clinical features of the patients studied.

Tumor ID	Tumor type	Hereditary PH/PGL type	Gene	Mutation	Sex	Age at surgery	Tumor size (mm)	Clinical characteristics	Familial characteristics
P11	PH	MEN2	RET	A883F	F	57	25	bilateral PH, MTC, marfanoid habitus	
P20	PH	MEN2	RET	C634R	F	29	32	bilateral PH, MTC, MEN-2 family's history	
P12	PH	neurofibromatosis type 1	NF1	ND	F	32	25	unique PH, “café-au-lait” spots, Lisch nodules, scoliosis	
P19	PH	neurofibromatosis type 1	NF1	ND	F	32	50	unique PH, “café-au-lait” spots, Lish nodules, neurofibroma	
P21	PH	neurofibromatosis type 1	NF1	ND	F	39	35	unique PH, “café-au-lait” spots, Lish nodules	
P9	EA FPGL	hereditary paraganglioma	SDHD	R22X	M	61	22	EA abdominal and bilateral carotid PGL	
P10	EA FPGL	hereditary paraganglioma	SDHB	L207fs	M	37	70	EA bladder PGL	
P22	PH	hereditary paraganglioma	SDHB	P56TyrfsX5	F	20	52	unique PH	
P23	PH	hereditary paraganglioma	SDHB	C253Y	F	21	110	unique PH	
P24	EA PGL	hereditary paraganglioma	SDHB	F238SerfsX10	F	10	40	unique PGL	
P25	PH	hereditary paraganglioma	SDHB	R46G	F	31	35	Unique PH	
P26	EA PGL	hereditary paraganglioma	SDHD	R22X	M	32	35	Thoracic PGL, carotid PGL	Father: bilateral carotid PGL; brother: bilateral neck PGL
P27	EA PGL	hereditary paraganglioma	SDHC	del exon 2	F	16	45	unique PGL	
P1	PH	von Hippel Lindau	VHL	Complete deletion	M	32	30	unique PH, cerebellar and bulbar HB	
P2	PH	von Hippel Lindau	VHL	S80I	M	33	30	bilateral PH, retinal and cerebellar HB, pancreatic kyst	
P8/16	EA FPGL	von Hippel Lindau	VHL	R161Q	F	24	30	multiple EA abdominal PGL, bilateral PH, renal kyst	
P6	PH	von Hippel Lindau	VHL	R167Q	M	26	55	unilateral PH, retinal and cerebellar HB, renal cancer	
P7	PH	von Hippel Lindau	VHL	L178P	M	15	30	bilateral PH, carotid PGL	P17's cousin; uncle: bilateral PH, renal cancers, bulbar HB
P14	PH	von Hippel Lindau	VHL	Y98H	F	29	30	bilateral PH	P18's daughter; aunt : unique PH, cervical and thoracic PGL
P17	PH	von Hippel Lindau	VHL	L178P	M	13	50	bilateral PH	P7's cousin; father : bilateral PH, renal cancers, bulbar HB
P18	PH	von Hippel Lindau	VHL	Y98H	M	51	30	unique PH, retinal HB, renal cancer	P14's father; sister : unique PH, cervical and thoracic PGL
P3	PH	von Hippel Lindau	VHL	Y156C	M	18	30	unique PH, no renal cancer, no HB	mother : unique PH, no renal cancer, no HB
P4	EA FPGL	von Hippel Lindau	VHL	Y156C	M	20	25	EA abdominal PGL, no renal cancer, no HB	mother: bilateral PH, vagal PGL, no renal cancer, no HB
P5	PH	von Hippel Lindau	VHL	P97L	F	17	30	unique PH, no renal cancer, no HB	brother : bilateral PH, no renal cancer, no HB
P13	PH	von Hippel Lindau	VHL	Y156H	F	65	40	bilateral PH, multiple EA PGL, no HB, no renal cancer	

PH/PGL: pheochromocytoma/paraganglioma; EA FPGL: extra-adrenal functional paraganglioma; MTC: medullary thyroid carcinoma; HB: hemangioblastoma

For microarray studies, 42 additional patients with hereditary PH/PGL were recruited for a total of 68 tumors studied (see Table 3 for clinical characteristics of patients).

**Table 3:** Clinical characteristics of the patients included in the microarray study

Characteristic	Subcategory	Number (n = 68)
**Age**	*Median (years)*	28
	*Range (years)*	7–76
**Gender**	*Male*	35
	*Female*	33
**Tumor location**	*Adrenal pheochromocytoma*	52
	*Abdominal paraganglioma*	14
	*Thoracic paraganglioma*	2
	*Lymph node metastasis*	3
**Malignancy**	*No*	53
	*Yes*	15
**Gene mutated**	*RET*	9
	*NF1*	9
	*SDHB*	17
	*SDHC*	2
	*SDHD*	3
	*VHL*	28

### Ethics Statement

Informed signed consent for germline and somatic DNA analysis was obtained from each patient recruited by the COMETE network, and the study was formally approved by an institutional review board (CPP Paris-Cochin).

### Immunohistochemistry

Paraffin blocks were cut and sections (6-micrometers thick) were mounted on Superfrost plus slides and used for immunohistochemistry as previously described [31]. The protocol involved a biotinylated secondary antibody (Vector Laboratories), an avidin-biotin-peroxidase complex (Vectastain ABC Elite; Vector Laboratories) and diaminobenzidin (Vector Laboratories) or Histogreen (Abcys) as chromogens for the peroxidase activity. Antibodies were as follows: HIF-1α (H1alpha67, abcam, 1/500), HIF-2α (ab199, abcam, 1/1000), CD34 (Clone QBEND 10, Immunotech, 1/100), α-actin (#M0851, Dako, 1/1000), SDHB (HPA002868, Sigma-Aldrich, 1/500), Glut1 (Labvision, 1/200), p53 (Ab-8, Neomarkers 1/1000), TIGAR (abcam, 1/200). p53 expression was revealed using the Tyramide Signal Amplification (TSA) System (Perkin Elmer). Heat-mediated antigen retrieval was performed for p53, Glut1, SDHB and TIGAR (10 mM citrate buffer, pH 6, 15 min), and for HIF-1α and HIF-2α (Tris 100 mM, EDTA 1 mM, 0,05% Tween, pH 9, 45 min).

### Quantification of Vascular Density

Vascular density was measured on sections after CD34 immunostaining on the two independent samples obtained from each patient. For each sample, total blood vessels were counted in eight randomly chosen fields of 0.125 mm^2^. For one patient (P6), vascular density could not be evaluated because of an anarchic vascular architecture.

### Western Blotting

Frozen tissues were lysed in protein extraction buffer (25 mM Tris, 100 mM NaCl, 0.5% NP40, 0.5% deoxycholic acid, 5 mM EDTA, protease inhibitor cocktail (Sigma)).

Aliquots of 50 µg of protein were separated on SDS-PAGE denaturing gels and transferred to either PVDF (Immobilon-P, Millipore) for SDHB, SDHA and OXPHOS experiments or nitrocellulose membranes (Hybond-ECL, Amersham Biosciences) for p53 and HXK-II experiments. Ponceau staining confirmed equivalent loading of each sample and membranes were then blocked in 5% milk in TBST buffer, and probed with the primary antibody.

Antibodies used for western blot experiments were as follows: SDHB (Molecular Probes, 1/1000), Oxphos complexes kit (MitoSciences, 1/200), SDHA (Molecular Probes, 1/1000), PDK-1 (#KAP-PK112, Stressgen, 1/2000), HXK-II (C-14, sc-6521, Santa Cruz Biotechnology, 1/200), p53 (Ab-8, Neomarkers 1/400) and β-Actin (Clone AC-74, Sigma-Aldrich, 1/5000). The membranes were then incubated with horseradish peroxidase-linked secondary antibodies (Amersham Biosciences, 1/5000), and bound antibodies were visualized using ECL Plus reagent (Amersham Biosciences).

### Assessment of Mitochondrial Respiratory Chain Function and Enzyme Assay

Activities of cytochrome *c* oxidase (COX; complex IV), succinate cytochrome *c* reductase (SCCR; complexes II+III), quinol cytochrome *c* reductase (QCCR; complex III) and lactate dehydrogenase were measured spectrophotometrically using a pseudo dual-wavelength Cary 50 spectrophotometer (Varian, Melbourne, Australia) [40].

### Microarray

Tumor samples (20 to 30 mg) were powdered under liquid nitrogen. RNAs were extracted using RNeasy mini kit (Qiagen). Aliquots of the RNA were analyzed by electrophoresis on a Bioanalyser 2100 (Agilent Technologies) and quantified using Nano Drop ND-1000 (Labtech). Stringent criteria for RNA quality were applied to rule out degradation, especially a 28 s/18 s ratio above 1.5. Microarray analyses were performed using 3 µg of total RNA of each sample as starting material and 10 µg cRNA per hybridization (GeneChip Fluidics Station 400; Affymetrix, Santa Clara, CA). The total RNA were amplified and labeled following the manufacturer's one-cycle target labeling protocol (http://www.affymetrix.com). The labeled cDNA were then hybridized to HG-U133 Plus 2.0 Affymetrix GeneChip arrays (Affymetrix). The chips were scanned with a GCOS 1.4.

### Statistical Analysis

Statistical analyses were performed using the Stat View software (SAS Institute Inc.). Differences were evaluated by ANOVA Bonferroni Test. A p value <0.05 was considered statistically significant. Microarray analyses were performed with R system software (http://www.R-project.org, V2.3.0) including packages of Bioconductor [41]. Raw feature data from Affymetrix HG-U133 Plus 2.0 GeneChip^TM^ microarrays were normalized using robust multi-array average (RMA) method (Bioconductor package *affy*) [42]. We used Welch's t-tests to identify genes differentially expressed between groups of samples (R package *stats*). Clustering were performed using Ward linkage and (1-Pearson coefficient of correlation) as inter-individual distance (R package *cluster*). Principal component analysis was performed using the function *prcomp* (R package *stats*).

Genes from the KEGG pathway ‘Oxidative phosphorylation’ (hsa00190) were obtained from (http://www.genome.ad.jp/kegg/kegg2.html). The glycolysis pathway genes comprised glucose transporters, glycolytic enzymes, lactate dehydohygenase and lactate transporter, as well as TIGAR.
